# Concomitant *TP53* mutations with response to crizotinib treatment in patients with *ALK*‐rearranged non‐small‐cell lung cancer

**DOI:** 10.1002/cam4.2043

**Published:** 2019-03-07

**Authors:** Peng Song, Fanshuang Zhang, Yan Li, Guangjian Yang, Wenbin Li, Jianming Ying, Shugeng Gao

**Affiliations:** ^1^ Department of Thoracic Surgery National Cancer Center/National Clinical Research Center for Cancer/Cancer Hospital, Chinese Academy of Medical Sciences and Peking Union Medical College Beijing China; ^2^ Department of Pathology National Cancer Center/National Clinical Research Center for Cancer/Cancer Hospital, Chinese Academy of Medical Sciences and Peking Union Medical College Beijing China; ^3^ Department of Medical Oncology National Cancer Center/National Clinical Research Center for Cancer/Cancer Hospital, Chinese Academy of Medical Sciences and Peking Union Medical College Beijing China

**Keywords:** *ALK*, crizotinib, non‐small‐cell lung cancer, survival, *TP53*

## Abstract

**Background:**

*TP53* mutations are the most prevalent mutations detected in non‐small‐cell lung cancer (NSCLC) and have been revealed as a negative prognostic biomarker of outcome. The impact of concomitant *TP53* mutations in *ALK*‐rearranged NSCLC remains uncertain.

**Methods:**

Tumor samples from 64 *ALK*‐rearranged NSCLC patients receiving crizotinib treatment were subjected to next‐generation sequencing (NGS) to identify *TP53* mutational status. The clinicopathologic features of the* TP53* mutations and its impact on the effect of crizotinib treatment were analyzed.

**Results:**

Among the 64 *ALK*‐rearranged patients, 15 (23.4%) patients showed a *TP53* mutation. Of these, six cases had disruptive mutations and nine with nondisruptive mutations. The objective response rate (ORR) and disease control rate (DCR) for *TP53* mutated patients were both significantly lower compared with those for* TP53* wild‐type patients (*p* = 0.003 and 0.023, respectively). A significantly shorter progression‐free survival (PFS) was found in *TP53* mutated patients compared with* TP53* wild‐type patients (*p* = 0.045). Nondisruptive *TP53 *mutations were associated with a shorter PFS in comparison with disruptive *TP53* mutations in *ALK‐*rearranged patients (*p* = 0.069). When nondisruptive *TP53* mutated patients were in comparison with *TP53* wild‐type patients, nondisruptive *TP53* mutations were associated with a significant reduced PFS (*p* = 0.003).

**Conclusions:**

*TP53* mutations, especially nondisruptive mutations, negatively affected the response to crizotinib and correlated with shorter PFS in *ALK‐*rearranged NSCLC patients.

## 1 INTRODUCTION

Recently, the landscape of treatments for non‐small‐cell lung cancer (NSCLC) has been changed by the development of molecular diagnosis and targeted therapies. Anaplastic lymphoma kinase (*ALK*) gene rearrangement defines a distinct molecular subtype of NSCLC and has been found in 3%‐7% of all NSCLC patients.^1^, ^2^, ^3^ Crizotinib, a potent tyrosine kinase inhibitor (TKI) of ALK, ROS1 and MET, was the first molecule inhibitor targeting ALK to be widely used in the clinic.^4^ Two Phase III trials, PROFILE 1007 and PROFILE 1014 confirmed the benefit of crizotinib over cytotoxic chemotherapy in advanced *ALK*‐rearranged NSCLC.^5^, ^6^

Despite excellent response rates and durable responses in some cases, most patients treated with ALK TKIs inevitably progress within 1‐2 years due to the acquired resistance.^5^, ^6^, ^7^, ^8^, ^9^, ^10^ Various mechanisms of acquired resistance to ALK TKI have been determined, which were summarized into two major classes: *ALK*‐dependent resistance (*ALK* secondary resistance mutations or amplification) and *ALK*‐independent resistance (activation of bypass tracks and lineage changes).^11^ In addition to acquired resistance, a small number (approximately 5%) of patients with *ALK*‐rearranged NSCLC treated with first‐line crizotinib have progressive disease as their best response due to intrinsic resistance.^6^, ^12^ However, mechanisms of intrinsic resistance are poorly understood, and this represents an important gap in the field of ALK TKI resistance.

Although *TP53* mutations has been reported to be associated with inferior response to EGFR‐TKIs and poor outcome in *EGFR*‐mutated NSCLC patients, the association between *TP53* mutations and the effect of crizotinib treatment in* ALK*‐rearranged NSCLC patients was still uncertain. The aim of the present study was to investigate the clinicopathologic characteristics of *TP53* mutation in *ALK‐*rearranged NSCLC and its association with the effect of crizotinib in *ALK*‐rearranged NSCLC patients.

## 2 MATERIALS AND METHODS

### 2.1 Patients and samples

We retrospectively analyzed 64 patients with *ALK*‐rearranged NSCLC treated with crizotinib at Cancer Hospital, Chinese Academy of Medical Sciences (CAMS, Beijing, China) between January 2011 and December 2016. Epidemiologic and clinicopathologic data were collected, including age, sex, smoking history, histological type, Karnofsky physical score (KPS), pathological stage, previous treatment regimens, response to crizotinib and outcomes. This study was approved by our institutional review board and ethics committee of Cancer Hospital, CAMS.

### 2.2 Targeted next‐generation sequencing

The tumor specimens of the 64 patients were formalin‐fixed, paraffin‐embedded tissues and were all enough for evaluating by NGS. All specimens were subjected to NGS of 56 cancer‐related genes with use of a kit (Burning Rock Biotech, Guangzhou, China) according to our NGS protocol as previously reported.^13^

### 2.3 Clinical response evaluation

Oral crizotinib was administered at a dose of 250 mg twice daily continuously (28‐day cycles) until progressive disease (PD) or unacceptable toxicity. Best clinical response to crizotinib treatment was classified on the basis of interval CT scans as complete response (CR), partial response (PR), stable disease (SD) or PD using standard Response Evaluation Criteria in Solid Tumors criteria version 1.1.^14^ Tumor assessments were performed independently by experienced radiologists every 6‐8 weeks until RECIST‐defined disease progression. The objective response rate (ORR) was defined as the sum of CR and PR The disease control rate (DCR) was calculated as the percentage of patients with CR, PR, and SD.

### 2.4 Statistical analysis

Progression‐free survival (PFS) was calculated from the date of initiating crizotinib treatment to disease progression. Overall survival (OS) was defined as the time from first diagnosis of stage IIIB/IV or postoperative recurrent until death. The association between clinicopathologic characteristics, response and *TP53* mutational status was tested by the Pearson's χ^2^ test or Fisher exact test, when appropriate. The survival curves were estimated using the Kaplan‐Meier method, and differences in survival were tested by the log‐rank test. Cox regression univariate analysis was used to generate survival hazard ratios and 95% confidence intervals. The data were analyzed using SPSS software (version 20.0 of SPSS, IBM, Armonk, NY, USA). Statistical significance was assumed at two‐sided *p* value <0.05.

## 3 RESULTS

### 3.1 Patient characteristics

Clinicopathologic characteristics of patients, type of *ALK* fusion, crizotinib treatment line, type of response to crizotinib and *TP53 *status are shown in Table 1. The majority of patients were female (54.7%) and never smokers (76.6%). Almost all patients were diagnosed with adenocarcinoma (98.4%). Fifty‐two patients (81.3%) harbored an *EML4‐ALK *fusion whereas 12 patients (18.7%) harbored other fusion partners for *ALK* including *PRKAR1A, KLC1,* *AFTPH *and* EPS15*. Of cases with* EML4‐ALK* fusion, the most common *EML4‐ALK* variant was variant 3a/b (E6:A20, 46.2%), followed by variant 1 (E13:A20, 25.0%), variant 2 (E20:A20, 19.2%) and Other rare variants (E14:A20 and E18:A20, 9.6%).

**Table 1:** The clinical characteristics of 64 patients with *ALK*‐rearranged NSCLC

Characteristics	No. of patients (*n* = 64)
Sex	
Male	29 (45.3%)
Female	35 (54.7%)
Age (years)	
Median	50
Range	24‐82
Smoking history	
Never	49 (76.6%)
Current/Former	15 (23.4%)
Histology	
Adenocarcinoma	63 (98.4%)
Nonadenocarcinoma	1 (1.6%)
Type of *ALK* fusion	
Non‐*EML4‐ALK*	12 (18.7%)
*EML4‐ALK*	52 (81.3%)
Variant 1	13 (25.0%)
Variant 2	10 (19.2%)
Variant 3a/b	24 (46.2%)
Other variants	5 (9.6%)
KPS	
70 to <90	34 (53.1%)
90‐100	30 (46.9%)
p Stage	
IIIB/IV	60 (93.7%)
Postoperative recurrent	4 (6.3%)
Crizotinib treatment line	
First	33 (51.6%)
≥Second	31 (48.4%)
Response to crizotinib	
CR	3 (4.7%)
PR	43 (67.2%)
SD	12 (18.7%)
PD	6 (9.4%)
ORR	46 (71.9%)
DCR	58 (90.6%)
*TP53* status	
Wild‐type	49 (76.6%)
Mutated	15 (23.4%)
Exon 5	3 (20.0%)
Exon 6	7 (46.7%)
Exon 7	2 (13.3%)
Exon 8	3 (20.0%)
Disruptive/nondisruptive	
Disruptive	6 (40.0%)
Nondisruptive	9 (60.0%)

CR, complete response; DCR, disease control rate; KPS, Karnofsky physical score; PD, progressive disease; PR, partial response; ORR, objective response rate; SD, stable disease.

### 3.2 *TP53* mutation

Out of the 64 *ALK*‐rearranged patients, 15 (23.4%) patients showed a *TP53* mutation: 20.0% were on exon 5, 46.7% on exon 6, 13.3% on exon 7, and 20.0% on exon 8 (Table 1). According to a previous report about differentiation of *TP53* mutations,^15^ we divided *TP53* mutations into two types, disruptive and nondisruptive, and observed six disruptive mutations and nine nondisruptive mutations. It is worth noting that all disruptive mutations except one were located in exon 6, whereas nondisruptive mutations evenly distributed in the four exons (Supplementary Table S1). Supplementary Table S2 shows associations between *TP53* mutations and clinical characteristics. Smoking was significantly associated with high frequency of *TP53* mutations (*p* = 0.021). No statistically significant association was found between *TP53* mutations and either sex, age, histology, KPS and tumor pathological stage.

### 3.3 *TP53* status in relation to* ALK* fusion

We found no significant association between *TP53* status and two types of *ALK* fusion (*EML4‐ALK* and Non‐*EML4‐ALK*) and different *EML4‐ALK* variants (Variant 1, Variant 2, Variant 3a/b) (Supplementary Tables S3 and S4). With regard to the distribution of *TP53* gene mutations, *TP53* exon 6 mutations and 8 mutations were both more common in patients with *EML4‐ALK *fusion. In addition, *TP53* disruptive mutations were more common in patients with *EML4‐ALK *variant 2 (Supplementary Table S4).

### 3.4 Response, PFS, and *TP53* mutations

ORR and DCR to crizotinib treatment in all 64 patients were 71.9% and 90.6%, respectively. The ORR and DCR for *TP53* wild‐type patients were both significantly higher compared with those for *TP53* mutated patients (*p* = 0.003 and 0.023, respectively; Table 2). Patients with nondisruptive *TP53* mutations showed lower ORR and DCR to crizotinib than patients with disruptive *TP53* mutations, although this difference was not statistically significant (*p* = 0.136 and 0.103, respectively; Table 2).

**Table 2:** ORR and DCR to crizotinib treatment in *ALK*‐rearranged NSCLC patients

	ORR	*p* value	DCR	*p*
Type of *ALK* fusion (*n = *64)		0.726		0.312
*EML4‐ALK*	38 (73.1%)		48(92.3%)	
Non‐*EML4‐ALK*	8 (66.7%)		10(83.3%)	
*EML4‐ALK* variants (*n = *49)		0.135		0.566
Variant 1	8 (61.5%)		12(92.3%)	
Variant 2	9 (90.0%)		10(100%)	
Variant 3a/b	19 (79.2%)		21(87.5%)	
Other *EML4‐ALK* variants	2 (40%)		5(100%)	
*TP53* status (*n = *64)		0.003		0.023
Mutated	6 (40.0%)		11(73.3%)	
Wild‐type	40 (81.6%)		47(95.9%)	
*TP53* mutation type (*n = *15)		0.136		0.103
Disruptive	4 (66.7%)		6(100%)	
Nondisruptive	2 (22.2%)		5(55.6%)	
*TP53* mutation site (*n = *15)		0.166		0.199
Exon 5	0		1(33.3%)	
Exon 6	4 (57.1%)		6(85.7%)	
Exon 7	0		1(50.0%)	
Exon 8	2 (66.7%)		3(100%)	

DCR, disease control rate; ORR, objective response rate.

**Figure 1:**
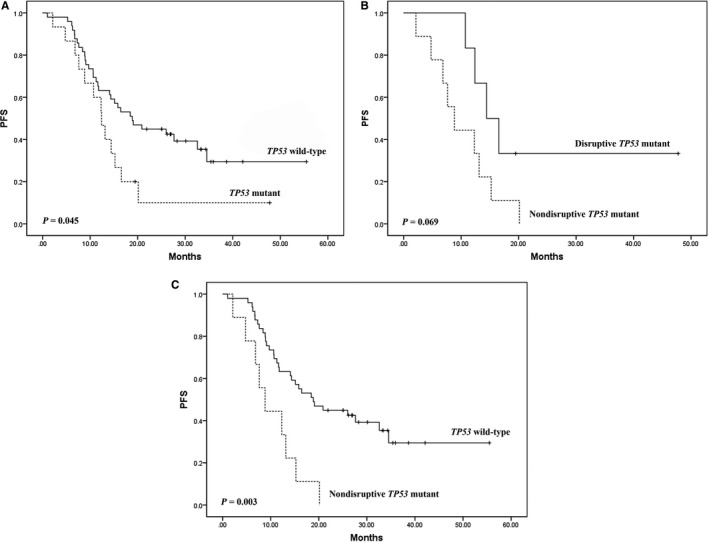
Kaplan‐Meier curves for progression‐free survival (PFS) for patients with anaplastic lymphoma kinase (*ALK*)‐rearranged non‐small‐cell lung cancer (NSCLC) who were treated with crizotinib according to TP53 mutation status. (A) PFS of *TP53* mutant patients compared with *TP53 *wild‐type patients. (B) PFS of nondisruptive *TP53* mutant patients compared with disruptive* TP53 *mutant patients. (C) PFS of nondisruptive *TP53* mutant patients compared with *TP53* wild‐type patients

Overall, median PFS and OS was 15.5 months (2.1‐55.5) and 48.8 months (11.8‐not reached). PFS was significantly longer in *TP53* wild‐type patients than in *TP53* mutated patients (HR = 1.96, 95% CI = 1.02‐3.80, *p* = 0.045; Figure 1A). Disruptive *TP53* mutations were associated with a longer PFS in comparison with nondisruptive *TP53* mutation in those with *ALK* rearrangements (HR = 2.91, 95% CI = 0.88‐9.63, *p* = 0.069; Figure 1B). When nondisruptive* TP53* mutated patients were in comparison with *TP5* wild‐type patients, nondisruptive *TP53* mutations were associated with a significant reduction in PFS (HR = 3.27, 95% CI = 1.51‐7.08, *p* = 0.003; Figure 1C). We found no significant difference in OS according to *TP53* mutation status.

## 4 DISCUSSION

In the current study, we explored the correlation between *TP53* mutations and the outcome of *ALK*‐rearranged NSCLC patients treated with crizotinib. Our data revealed that *TP53* mutations were significantly associated with reduced response to crizotinib and inferior PFS. In particular, nondisruptive *TP53* mutations represent a heterogeneous subgroup of *ALK*‐rearranged NSCLC patients with inferior PFS. To our best knowledge, we investigate for the first time the association between nondisruptive *TP53* mutations and survival in a case series of *ALK*‐rearranged NSCLC patients.

Mutations of* TP53 *gene are the most prevalent mutations detected in lung cancer and often coexist with driver mutations. *TP53* mutations are present in almost half of NSCLC patients and the incidence of *TP53* mutations is higher in lung squamous cell carcinomas than that in lung adenocarcinomas, with mutation rates between 20% and 40% in the latter.^16^, ^17^, ^18^ In line with prior studies, we found that *TP53* mutations occurred in 23.4% of *ALK*‐rearranged NSCLC patients in our case series. In terms of a previous study, it is the direct mutagenic action on DNA the means by which smoking causes lung cancer and the *TP53* gene is one of the most frequent targets of tobacco smoking‐related DNA mutations.^19^ The findings of our study showed that smoking was significantly associated with high frequency of *TP53* mutations, consistent with several studies in which *TP53* mutations more frequently occurred in patients with smoking‐associated cancer (26%‐71%) compared with patients who never smoked (8%‐47%).^20^, ^21^, ^22^

As a pivotal tumor suppressor, p53 regulates a series of cell activities to protect against cancer. In response to various types of cellular stress, TP53 gene is activated, resulting in the accumulation of p53 protein, which is implicated in cell‐cycle arrest, DNA repair, senescence, apoptosis, metabolism, aging, and differentiation.^23^ The total biological function of these processes is to prevent the transformation of a normal cell into a cancerous cell.^14^ Therefore, the transforming potential of oncogenes can be accelerated by loss of p53 function which mainly originates from *TP53* mutations. *TP53* mutation is one of the most widely investigated prognostic biomarker in patients with NSCLC. In unselected NSCLCs, prognostic impact of* TP53* mutations on survival remains controversial.^24^, ^25^, ^26^, ^27^, ^28^ In *EGFR‐*mutated NSCLCs, previous studies have suggested that *TP53* mutations were not only associated with poor response to EGFR TKIs, but also correlated with shorter survival in these patients.^29^, ^30^, ^31^, ^32^ However, the results of aforementioned studies only partly reached statistical significance. In *ALK*‐rearranged NSCLCs, Kron et al performed a detailed analysis of concomitant mutations and revealed that the existence of concomitant *TP53* mutations was an adverse prognostic factor for PFS to ALK TKIs and a negative predictor for OS.^33^ In the present study, we found that the occurrence of *ALK *rearrangement with concomitant* TP53* mutations negatively affected the response to crizotinib and correlated with shorter PFS, which is consistent with previous findings.

Moreover, we divide* TP53* mutations into two types, disruptive and nondisruptive, based on the degree of disorder of p53 protein structure predicted from the crystal structure of the p53–DNA complexes.^15^ The results of our study demonstrated that nondisruptive *TP53* mutations represent a heterogeneous subgroup of *ALK*‐rearranged NSCLC patients with inferior PFS. When nondisruptive *TP53* mutated patients were in comparison with *TP53* wild‐type patients, nondisruptive *TP53* mutations were associated with a significant reduction in PFS (*p* = 0.003). Although a correlation between nondisruptive *TP53* mutations and worse outcomes has been reported in *EGFR*‐mutated lung NSCLCs,^34^ this study first reported the negative prognostic role of nondisruptive *TP53* mutations in *ALK‐*rearranged NSCLCs treated with crizotinib. The mechanism underlying the negative prognostic role of nondisruptive *TP53* mutations has not been fully elucidated. Prior experimental evidence revealed that nondisruptive mutations caused partial loss of p53 functions, whereas, of note, the retained functional properties of p53 protein were often associated with gain‐of‐function (GOF) activities that exerted by abrogating the function of p53‐related proteins p63/p73 and modulating transcriptional output.^15^, ^35^, ^36^, ^37^, ^38^ Mutant p53 GOF activities can render some cell types increased tumorigenicity, motility, and growth rate.^39^, ^40^ Moreover, increased metastasis and invasiveness and decreased sensitivity to chemotherapeutic drugs are features of mutant p53 GOF activities, which have been demonstrated in cell models.^41^, ^42^

There are some limitations in the current study. First, it was a retrospective, single institutional study and therefore patient selection bias was inevitable. Second, due to a relatively small cohort, the results cannot be regarded as definitive. A prospective study with a larger sample size of *ALK*‐rearranged NSCLCs with *TP53* mutations is warranted in the future.

Conclusively, our results highlighted the negative prognostic role of *TP53 *mutations in* ALK‐*rearranged NSCLC patients treated with crizotinib. Moreover, nondisruptive *TP53* mutations seem to represent a heterogeneous subgroup of* ALK‐*rearranged NSCLC patients with inferior PFS.

## CONFLICT OF INTEREST

The authors declare no potential conflicts of interest.
