# Supplementary material for: Concomitant TP53 mutations with response to crizotinib treatment in patients with ALK‐rearranged non‐small‐cell lung cancer
Source: Cancer Med. 2019 Mar 7;8(4):1551–7. doi: 10.1002/cam4.2043 (PMC6488212; doi:10.1002/cam4.2043)
Supplement: Supplementary file 1 [file CAM4-8-1551-s001.docx]

**Supplementary Table 1.** Distribution of disruptive and non-disruptive mutations in the different *TP53* mutation sites

|  | **Disruptive (*n* = 6)** | **Non-disruptive (*n* = 9)** | **All (*n* = 15)** | **P** |
| --- | --- | --- | --- | --- |
| ***TP53* mutation sites** |  |  |  | 0.099 |
| Exon 5 | 0 | 3 (33.3%) | 3 (20%) |  |
| Exon 6 | 5 (83.3%) | 2 (22.2%) | 7 (46.7%) |  |
| Exon 7 | 0 | 2 (22.2%) | 2 (13.3%) |  |
| Exon 8 | 1 (16.7%) | 2 (22.2%) | 3 (20%) |  |

**Supplementary Table 2.** Relationship of *TP53* mutations and characteristics of patients

| **Characteristics** | **No. of Patients**  **(*n* = 64)** | ***TP53* mutant**  **(*n* = 15)** | ***TP53* wild-type**  **(*n* = 49)** | **P** |
| --- | --- | --- | --- | --- |
| **Sex** |  |  |  | 0.559 |
| Male | 29 (45.3%) | 8 (53.3%) | 21 (42.9%) |  |
| Female | 35 (54.7%) | 7 (46.7%) | 28 (57.1%) |  |
| **Age** (years) |  |  |  | 0.556 |
| < 50 | 32 (50.0%) | 9 (60.0%) | 23 (46.9%) |  |
| ≥50 | 32 (50.0%) | 6 (40%) | 26 (53.1%) |  |
| **Smoking history** |  |  |  | 0.021 |
| Never | 46 (71.9%) | 7 (46.7%) | 39 (79.6%) |  |
| Current/Former | 18 (28.1%) | 8 (53.3%) | 10 (20.4%) |  |
| **Histology** |  |  |  | 0.234 |
| Adenocarcinoma | 63 (98.4%) | 14 (93.3%) | 49 (100%) |  |
| Nonadenocarcinoma | 1 (1.6%) | 1 (6.7%) | 0 |  |
| **KPS** |  |  |  | 1.000 |
| 70–< 90 | 34 (53.1%) | 8 (53.3%) | 26 (53.1%) |  |
| 90–100 | 30 (46.9%) | 7 (46.7%) | 23 (46.9%) |  |
| **p Stage** |  |  |  | 0.565 |
| IIIB/IV | 60 (93.7%) | 15 (100%) | 45 (91.8%) |  |
| Postoperative recurrent | 4 (6.3%) | 0 | 4 (8.2%) |  |

Abbreviations: KPS: Karnofsky Physical score.

**Supplementary Table 3.** Correlation between *TP53* and *ALK* rearrangements

|  | **Type of *ALK* fusion** | | **P** |
| --- | --- | --- | --- |
|  | ***EML4-ALK*** | **Non *EML4-ALK*** |  |
| ***TP53* status** (n=64) |  |  | 1.000 |
| Mutated | 12 (23.1%) | 3 (25%) |  |
| Wild-type | 40 (76.9%) | 9 (75%) |  |
| ***TP53* mutation type** (n=15) |  |  | 0.229 |
| Disruptive | 6 (50.0%) | 0 |  |
| Non-disruptive | 6 (50.0%) | 3 (100%) |  |
| ***TP53* mutation sites** (n=15) |  |  | 0.052 |
| Exon 5 | 1 (8.3%) | 2 (66.7%) |  |
| Exon 6 | 7 (58.3%) | 0 |  |
| Exon 7 | 1 (8.3%) | 1 (33.3%) |  |
| Exon 8 | 3 (25.0%) | 0 |  |

**Supplementary Table 4.** Association between *TP53* and *EML4-ALK* variants

|  | ***EML4-ALK* Variant 1** | | **P** | ***EML4-ALK* Variant 2** | | **P** | ***EML4-ALK* Variant 3a/b** | | **P** |
| --- | --- | --- | --- | --- | --- | --- | --- | --- | --- |
|  | Yes | No |  | Yes | No |  | Yes | No |  |
| **TP53 status** (n=52) |  |  | 0.466 |  |  | 0.420 |  |  | 1.000 |
| Mutated | 4 (30.8%) | 8 (20.5%) |  | 1 (10.0%) | 11 (26.2%) |  | 6 (25.0%) | 6 (21.4%) |  |
| Wild-type | 9 (69.2%) | 31 (79.5%) |  | 9 (90.0%) | 31 (73.8%) |  | 18 (75.0%) | 22 (78.6%) |  |
| **TP53 mutation** (n=15) |  |  | 0.545 |  |  | 1.000 |  |  | 0.567 |
| Disruptive | 3 (75.0%) | 3 (37.5%) |  | 1 (100%) | 5 (45.5%) |  | 2 (33.3%) | 4 (66.7%) |  |
| Non-disruptive | 1 (25.0%) | 5 (62.5%) |  | 0 | 6 (54.5%) |  | 4 (66.7%) | 2 (33.3%) |  |
